# Regulatory T cell phenotype and anti-osteoclastogenic function in experimental periodontitis

**DOI:** 10.1038/s41598-020-76038-w

**Published:** 2020-11-04

**Authors:** Carla Alvarez, Salwa Suliman, Rawan Almarhoumi, Maria Elena Vega, Carolina Rojas, Gustavo Monasterio, Mario Galindo, Rolando Vernal, Alpdogan Kantarci

**Affiliations:** 1 The Forsyth Institute, Cambridge, MA USA; 2 Periodontal Biology Laboratory, Faculty of Dentistry, Universidad de Chile, Santiago, Chile; 3 Department of Clinical Dentistry, Center for Clinical Dental Research, University of Bergen, Bergen, Norway; 4 Program of Cellular and Molecular Biology, Institute of Biomedical Sciences (ICBM), Faculty of Medicine, Universidad de Chile, Santiago, Chile; 5 Millennium Institute on Immunology and Immunotherapy, Faculty of Medicine, University of Chile, Santiago, Chile

**Keywords:** Chronic inflammation, Cellular immunity, Regulatory T cells, T-helper 17 cells, Periodontitis

## Abstract

The alveolar bone resorption is a distinctive feature of periodontitis progression and determinant for tooth loss. Regulatory T lymphocytes (Tregs) display immuno-suppressive mechanisms and tissue repairing functions, which are critical to support periodontal health. Tregs may become unstable and dysfunctional under inflammatory conditions, which can even accelerate tissue destruction. In this study, experimental periodontitis was associated with the progressive and increased presence of Th17 and Treg-related mediators in the gingiva (IL-6, IL-17A, IL-17F, RANKL, IL-10, TGF-β and GITR; *P* < 0.05), and the proliferation of both Treg and Th17 cells in cervical lymph nodes. Tregs from cervical lymph nodes had reduced Foxp3 expression (> 25% MFI loss) and increased IL-17A expression (> 15%), compared with Tregs from spleen and healthy controls. Tregs gene expression analysis showed a differential signature between health and disease, with increased expression of Th17-associated factors in periodontitis-derived Tregs. The ex vivo suppression capacity of Tregs on osteoclastic differentiation was significantly lower in Tregs obtained from periodontally diseased animals compared to controls (*P* < 0.05), as identified by the increased number of TRAP^+^ osteoclasts (*P* < 0.01) in the Tregs/pre-osteoclast co-cultures. Taken together, these results demonstrate that Tregs become phenotypically unstable and lose anti-osteoclastogenic properties during experimental periodontitis; thus, further promoting the Th17-driven bone loss.

## Introduction

Periodontitis is a complex multifactorial disease characterized by the deregulated host immune response against the dysbiotic polymicrobial community colonizing the periodontal environment^1^. The alveolar bone resorption is a direct outcome of the chronic inflammatory process and one of the main features of the disease progression^2^. Osteoclasts are the central bone-resorptive cell, which differentiates from monocyte/macrophage precursors after local stimulation with crucial factors, such as receptor activator of nuclear factor κB ligand (RANKL) and the macrophage colony-stimulating factor (M-CSF)^3^.

The imbalance between bone resorption and formation relies on the antagonistic relationship between effector immune cells, led by the activity of interleukin (IL)-17A-expressing T-helper type 17 (Th17) cells^4^ and immunosuppressive cells such as T regulatory (Treg) lymphocytes^5^. Thus, the alveolar bone resorption is, in part, a consequence of the Th17/Treg imbalance in periodontal tissues, which in turn causes the upregulation of RANKL, and other proinflammatory cytokines, that further promote bone loss^6^.

Tregs are a subset of CD4^+^ T lymphocyte with immunosuppressive functions that contribute to the maintenance of peripheral tolerance, prevention of autoimmunity, and limitation of chronic inflammation. Tregs express their signature transcription factor forkhead box P3 (Foxp3) and interleukin-2 receptor alpha chain, termed CD25^7^. Their suppressive functions are mainly due to regulating the effector functions of proinflammatory cells, such as T and B cells, neutrophils, and macrophages^7^. In periodontal tissues, Tregs are essential for the maintenance of periodontal health by controlling periodontal inflammation, soft tissue breakdown, and alveolar bone resorption^8^.

Even though Tregs are terminally differentiated, they may lose their phenotype and suppressive functions in a chronic inflammatory milieu^9^. The maintenance of the Treg phenotype has multiple clinical implications, especially in the context of bone-resorptive pathologies and the development of new therapies. Recent studies have shown that Tregs can become unstable and dysfunctional under inflammatory conditions and may foster the pathogenesis of different diseases^10^. The immune regulatory features of Tregs depend on the sustained high expression of Foxp3, which can be altered, among others, by epigenetic changes in the Treg-specific demethylated zone (TSDR) located in the *foxp3* loci, thus modifying their overall transcriptome. In this context, the reduced expression of the canonic Treg genetic program may determine their loss of function and eventually promote the gain of Th17-related functions, such as the production of IL-17A^11,12^, particularly in the presence of sustained IL-6 and TGF-β stimuli^13^.

The chronic inflammatory process associated with periodontitis has been linked to the enrichment of exFoxp3Th17 cells within the gingival tissues, indicating the full transdifferentiation of Tregs into Th17 cells; however, the impact of periodontal inflammation on Treg phenotype and function, and their temporal relationship with the alveolar bone loss, have not been elucidated^14,15^. In this study, we hypothesized that chronic periodontal inflammation promotes the gain of Th17-related functions on Tregs, by modifying their phenotype and function in terms of their suppressive capacity over osteoclast differentiation and without the complete loss of Foxp3 expression. We expected these changes to be driven by lessened mRNA expression of Foxp3 due to the methylation of the Treg specific demethylated zone (TSDR) in the *foxp3* locus.

## Methods

### Animals and experimental periodontitis model

C57BL/6 wild type (WT) and B6.129(Cg)-Foxp3tm3(DTR/GFP)Ayr/J (Foxp3^DTR^) mice were purchased from the Jackson Laboratory (Maine, USA). For all experiments, 8–10 weeks old male and female littermate animals were used. Mice were maintained in a pathogen-free environment, 12:12 h light/dark cycle at 24 ± 0.5 °C, and 40–70% of relative humidity. The experimental protocols were approved by the Institutional Animal Care and Use Committee of the Forsyth Institute and the Bioethics Institutional Committee of the Dentistry Faculty, Universidad de Chile adhering to all local, national and international regulations and conventions, and standard scientific and ethical practices. All methods were performed in accordance with relevant guidelines and regulations.

Silk ligatures (6-0) were tied around maxillary second molars on both left and right jaws for 5, 10, 15, or 20 days, as previously described^16^. The alveolar bone loss was analyzed after defleshing and splitting the maxilla at the midline between the central incisors. One half was taken for scanning electron microscopy (SEM) analysis and the other for microcomputed tomography (μCT) analysis, as previously described^17^. The μCT imaging was done in a SkyScan 1278 instrument (Bruker), using the following parameters: 59 kV, 588 μA, 0.5° rotation, and 360° of angular range. The images were reconstructed using the NRecon software Version 1.10.7 (Bruker). All images were oriented to parallel the cement-enamel junction (CEJ) from the first and the second molar to the horizontal axis and making sure that both CEJ and root apex appeared in the same slice. A standardized region of interest (ROI) with the following anatomical limits was generated: furcation roof, root apex of both first and second molar, medial root surface of the first molar, and the distal root surface of the second molar (Data-viewer software version 1.5.0.0; Bruker). For SEM analysis, the samples were dehydrated through immersion in the following concentrations of ethanol: 50%, 70%, 95%, and 100%; then sputter-coated with gold layer to a thickness of 200 nm, and examined in a Jeol JSMIT300LV SEM (Jeol) at an accelerating voltage of 20 kV. Bone loss was measured at 30x. All data were analyzed by a single blinded observer.

### RNA extraction and quantitative real-time reverse transcriptase PCR (qRT-PCR)

Gingival samples were collected by making a vertical incision anterior to the upper maxillary first molars and posterior to the third molars, then a horizontal incision at the border of the gingiva (white collar of tissue surrounding teeth), transported in RNAlater (Life Technologies) and lysed using glass tissue homogenizers and 1 ml of Trizol (Invitrogen). The soluble fraction was then incubated for 10 min at 4 °C, and 200 μl of chloroform was added and incubated for 10 min at 4 °C. After centrifuging at 12,000 g for 20 min, the aqueous phase was collected, and the total RNA was precipitated in 500 μl of isopropyl alcohol and 20 μg/μl of glycogen (Roche) for 30 min. Then, all samples were centrifuged at 12,000 g for 20 min at 4 °C, and the RNA precipitate was washed once with 1 ml 75% ethanol. On the other hand, the total cytoplasmic RNA from Tregs isolated from WT animals was obtained using 400 μl of ice-cold lysis buffer containing 0.5% Igepal CA-630 (Sigma-Aldrich), 50 mM Tris-HCl (pH8), 100 mM NaCl and five mM MgCl_2_, as described previously^18^. All purified RNA samples were resuspended in 30 μl of sterile RNase-free milli-Q water and quantified in a Synergy HT spectrophotometer (Bio-Tek).

The first-strand of cDNA was synthesized from 1 μg of RNA using the reverse transcription kit SuperScript III (Invitrogen), according to the manufacturer's instructions. Quantitative real-time PCR amplification of cDNAs (50 ng) was performed using KAPA SYBR Fast qPCR kit (KAPA) and mouse gene-specific primers (Supplementary Figure 1b), for quantitation in a StepOne Real-Time PCR System (Applied Biosystems). All transcript levels were normalized to transcript levels of mouse18S rRNA. The data is presented as a fold-change of relative quantities using the 2^−ΔΔCt^ method.

**Figure 1:**
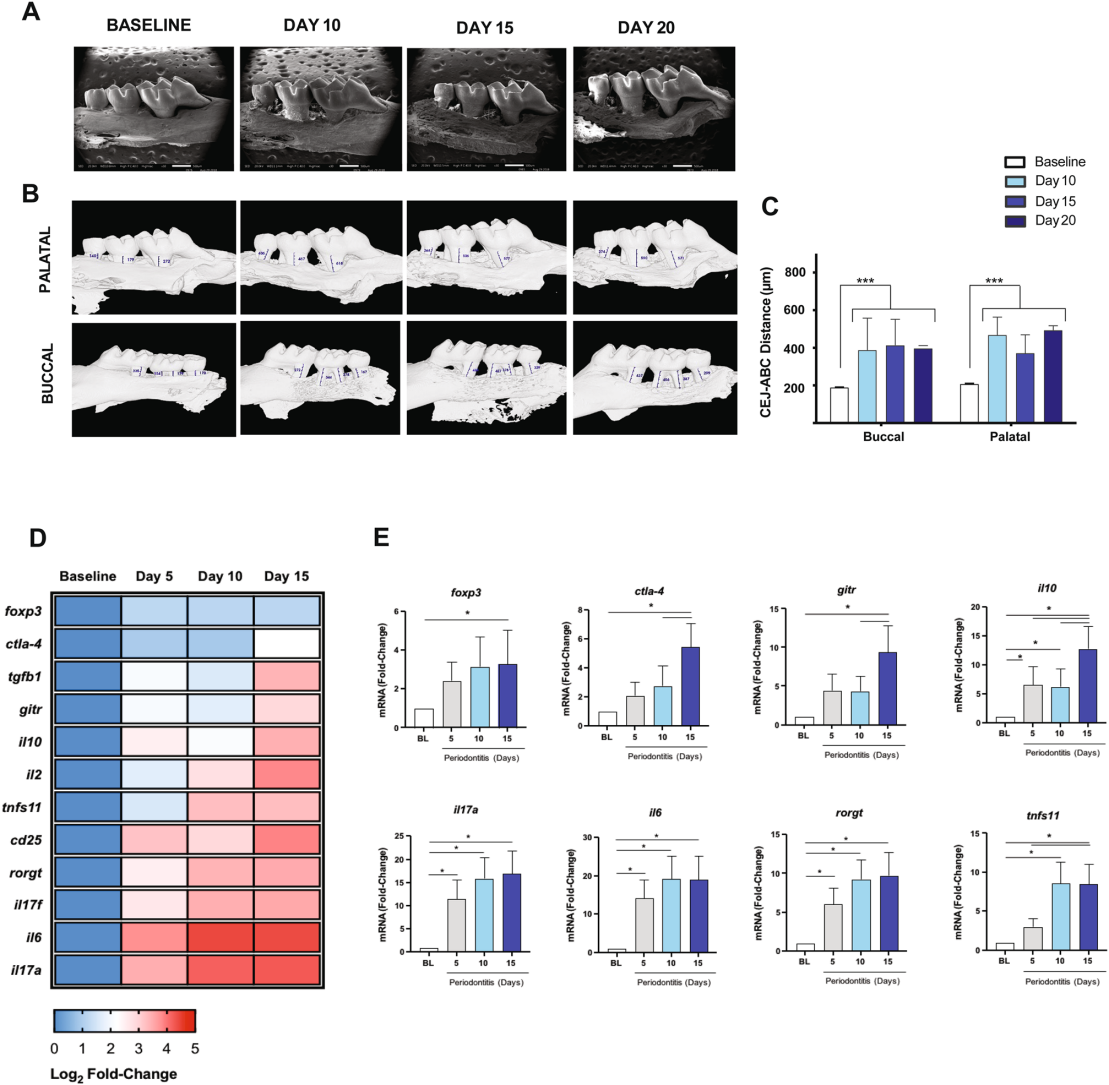
Bone loss and expression profile of Th17 and Treg markers in periodontal lesions. (**A**) The palatal vision of the upper right jaws by SEM. (**B**) The buccal and palatal vision of the right upper jaws with linear measurements between the cement-enamel junction (CEJ) and alveolar bone crest (ABC) by µCT. (**C**) The linear distance between CEJ and the ABC measured at days 10, 15, and 20 post-ligations and control animals (baseline). (**D**) Heat-map visualization of the log2 fold-change mean value (n = 4). (**E**) mRNA fold-change levels of cytokines, transcription factors, and membrane receptors associated with Th17 and Treg profiles in periodontal lesions of animals with ligature-induced periodontitis and control animals (BL). *P < 0.05, ***P < 0.001.

### Flow cytometry analysis of Tregs and Th17 cells

Single-cell suspensions were obtained from cervical lymph nodes that drain periodontal tissues, and spleens using 70 μm cell strainers (Sigma-Aldrich) rinsed-out with phosphate-buffered saline (PBS) containing 5% fetal bovine serum (FBS). For cytokine detection, 2–4 × 10^6^ cells were incubated for 4 h in RPMI -1640 (Gibco) supplemented with 10% FBS, 1% penicillin–streptomycin (Sigma), Brefeldin A (eBioscience), 50 ng/ml PMA (Sigma), and 1 μg/ml Ionomycin (Sigma). Cells were then washed with PBS and stained with the zombie UV™ Fixable Viability Kit (Biolegend) for 30 min in the dark. The extracellular staining was performed in PBS containing 5% FBS, using the following antibodies: anti-CD4 (GK1.5, Biolegend) and CD25 (PC61, Biolegend), for 30 min at 4 °C in the dark. The intracellular staining was done using a Fixation/Permeabilization staining kit following the manufacturer instructions (eBioscience) and using the following antibodies: anti-Foxp3 (MF-14, Biolegend), Rorγt (Q21-559, eBiosciences), and IL-17A (9B10, Biolegend). Cells were analyzed on a BD FACSCanto cytometer (BD Biosciences) using a sequential gating strategy according to the FSC/SSC and SSC/SSC parameters, live/dead staining, and CD4/CD25 markers. Data analysis was completed using the FlowJo software (version 10.6.2). For each animal, the experiments were performed separately.

### Isolation of Tregs and bone marrow-derived monocytes

Tregs were isolated from both wild-type and Foxp3^DTR^ mice (Foxp3^DTR^ also expresses eGFP). The cervical lymph nodes were harvested from periodontitis-affected animals and healthy controls. A single-cell suspension was obtained using a 70 μm cell strainer rinsed-out with PBS 5% FBS. From wild-type animals, the CD4^+^CD25^+^ cells were isolated using a microbeads-based regulatory T cell isolation kit (Miltenyi Biotec) by a two-step process: first, enrichment of CD4^+^ cells by cell depletion and second purification of CD25^+^ cells by positive cell selection. This two-step protocol ensures an increased CD4^+^CD25^+^ cell purity^19^. The phenotype of the obtained population was confirmed by flow cytometry (Supplementary Fig. 1a). From Foxp3^DTR^ mice, the CD4^+^ population was enriched from the single-cell suspension using the microbeads-based CD4^+^ T cell isolation kit (Miltenyi Biotec). Then, the cells were stained with CD4 and CD25 fluorochrome-conjugated antibodies, and the CD4^+^CD25^+^Foxp3^+^ cells were sorted on a FACSAria III (BD Biosciences) (Supplementary Figure 1b).

Bone marrow-derived monocytes (BMM) were obtained from C57BL/6 healthy mice. Femurs and fibulas were defleshed and transported in a conical-tube with 10 mL of PBS supplemented with 2% penicillin–streptomycin. The bone marrow was flushed out of the bones using PBS and a 10 mL syringe with a 24G needle. The samples were centrifuged at 300 × *g* for 5 min, the pellet resuspended in 1 ml of RBC lysis buffer (0.15 M NH_4_Cl, 10 mM KHC0_3_, and 0.1 mM EDTA in ddH_2_0) and incubated at room temperature for 2 min. PBS was added up to 50 mL, and the cell suspension was filtered through a 70 µm cell strainer. The resulting samples were spun-down, and the pellet resuspended in PBS 5% FBS to stain them with a fluorochrome-conjugated anti-CD11b antibody (M1/70, Biolegend). Finally, CD11b^+^ cells were sorted on a FACSAria III (BD Biosciences).

### Epigenetic analysis of the *foxp3* locus

CpG methylation analysis of four CpG residues for the *Foxp3 loci*, located at -2238 to -2207 from ATG in the Intron 1 (TSDR Region), was determined by pyrosequencing of bisulfite-modified genomic DNA from Tregs isolated from WT animals (> 90% enriched). The methylation analysis was conducted by EpigenDx (Assay ADS568FS2), as previously described^20^. The percentage of methylation for each GpG residue are shown separately.

### Osteoclast differentiation and Treg co-culture

The Treg and BMM co-culture setting was prepared, as previously described^21^. Briefly, purified CD11b^+^ monocytes (2.5 × 10^5^ cells/well) and CD4^+^CD25^+^Foxp3^+^ Tregs (5 × 10^4^ cells/well) from periodontitis-induced animals (pTregs) or baselines, animals without ligatures, controls (bTregs) were co-cultured in flat-bottom 96-well plates using Alpha-MEM media (Gibco) supplemented with 10% FBS and 1% penicillin–streptomycin in the presence of 30 ng/ml M-CSF and 50 ng/ml rmRANKL (R&D Systems) for 4 days. At day 2 of co-culture, fresh 30 ng/ml M-CSF and 50 ng/ml RANKL was added. CD11b^+^ monocytes (2.5 × 10^5^ cells/well) without Tregs were used as controls (BMM). All Tregs were pre-activated for 1 h before adding them to the co-cultures, with five µg/ml of soluble anti-CD3 antibody (17A2, R&D Systems). Osteoclast differentiation was evaluated by TRAP staining using a leukocyte acid phosphatase kit (Sigma-Aldrich). TRAP^+^ cells with three or more nuclei were considered osteoclasts and quantified using visible light microscopy (Axiostar Plus; Carl Zeiss Co.). For the analysis of Treg suppressive capacity on osteoclast differentiation, the number of TRAP^+^ osteoclasts in wells with Tregs (pTregs and bTregs) were normalized against the mean of their experimental control (BMM). Likewise, the Treg percentage of suppression was determined by considering the number of TRAP^+^ osteoclasts in BMM controls for each independent experiment as 100% differentiation capacity. Finally, the cell area for every identified TRAP^+^ osteoclast was analyzed using images at 40 × and the Fiji Image J software (Version 1.51).

### Statistical analysis

Data were analyzed using Graph Pad Prism software (Version 8.4.2). The normality of data distribution was determined using the Shapiro–Wilk test. Unpaired *t*-Student or *U*-Mann–Whitney tests were used to determine differences between two experimental groups. When more than two groups were compared, ANOVA or Kruskal–Wallis tests, followed by multiple comparison Tukey or Dunn post-hoc tests were applied. *P* values < 0.05 were considered statistically significant.

## Results

### Experimental periodontitis induces alveolar bone loss and Th17/Treg imbalance

To establish at which time-point (5, 10, 15, and 20 days) of periodontitis progression, the Th17/Treg imbalance occurs, we first analyzed both the periodontal inflammation and bone loss. After ten days, the bone loss around the first to third molars was evident and did not further progress for up to 20 days (Fig. 1). We observed a similar pattern of bone loss at both palatal and buccal sides of the maxilla (Fig. 1). At the gingival tissues, a significant increase in the mRNA levels of Th17-related genes -such as IL-6, IL-17A, and Rorγt- were detected early at five days, and reaching its maximum at ten days post-periodontitis induction (≈ 10–20 fold-change) (Fig. 1D,E). Similarly, the RANKL mRNA levels (tnfs11) increased significantly after 10- and 15-days post-periodontitis induction (≈ 10-change) (Fig. 1E). Interestingly, maximal expression of Treg-related genes Foxp3, IL-2, IL-10, CTLA-4, CD25, and GITR was reached later than Th17-related genes, on day 15 post-periodontitis induction (Fig. 1E, and Supplementary Figure 2). Moreover, significant but slight changes in levels of Foxp3 transcripts were only detected at day 15. These results suggest an interplaying between the functional balance of Th17/Tregs cell responses and bone resorption during the progression of periodontal disease.

**Figure 2:**
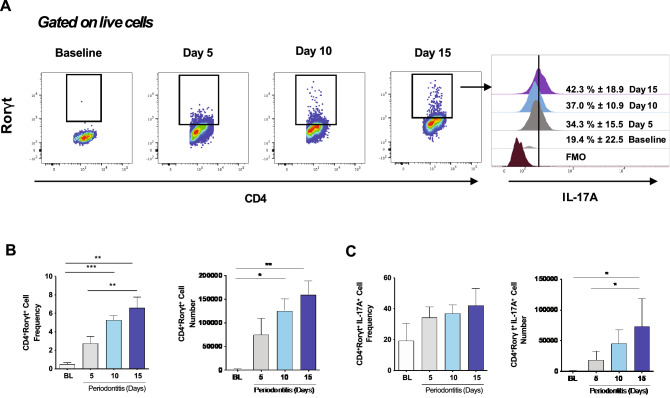
Th17 cells and IL-17A expression in cervical lymph nodes of animals with periodontitis. (**A**) Representative data of 4 independent experiments by flow cytometry with a mean percentage ± SD values. (**B**) Analysis of the frequency and the total number of CD4^+^Rorγt^+^ and CD4^+^Rorγt^+^IL-17A^+^ cells. FMO: Fluorescence minus one control. *P < 0.05, **P < 0.01, ***P < 0.001.

### Th17 cells are increased in cervical lymph nodes during experimental periodontitis

Since our primary goal was to assess the impact of experimental periodontitis on Tregs at cervical lymph nodes, we first sought to analyze whether periodontal inflammation induces changes in Th17 cells. We detected a significant increase of both the percentage of CD4^+^Rorγt^+^ cells within the CD4^+^ compartment and the total number of CD4^+^Rorγt^+^ cells at days 10 and 15 post-periodontitis induction (Fig. 2A,B). Besides, we observed the enrichment of total CD4^+^Rorγt^+^IL-17A^+^ cells at day 15, although the percentage of IL-17A-producing CD4^+^Rorγt^+^ cells remained constant (≈ 37%) (Fig. 2C). These results demonstrated an increase in the Th17-mediated response in cervical lymph nodes, which was associated with higher overall cellularity and increased organ size (Supplementary Figure 3) and early detection of Th17-related phenotypic markers temporally paired to periodontal inflammation.

**Figure 3:**
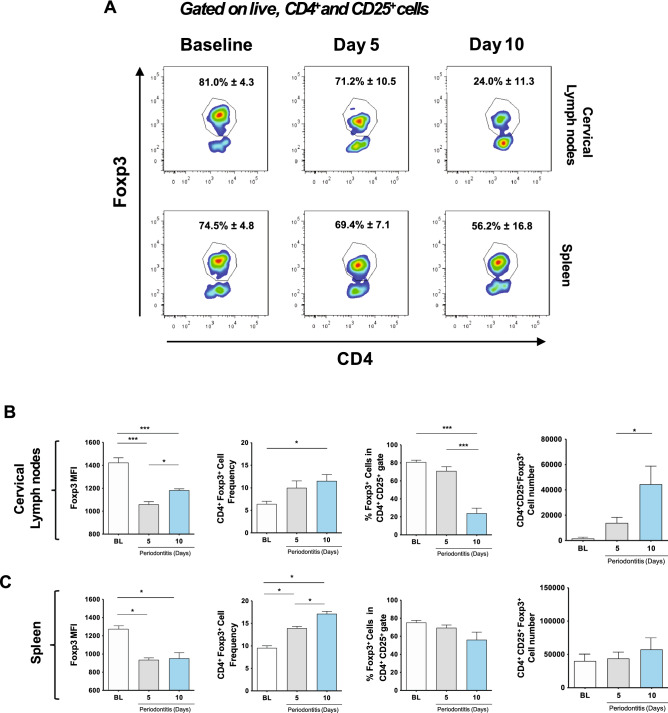
Time-dependent changes in Tregs in cervical lymph nodes and spleen during periodontitis progression. (**A**) Representative data of CD4^+^ Foxp3^+^ cells in CD4^+^CD25^+^ gate with mean ± SD values. Foxp3 Mean Fluorescence Intensity (MFI), frequency, and the total number of Foxp3^+^ cells in CD4^+^ and CD4^+^CD25^+^ gates in (**B**) cervical lymph nodes and (**C**) spleen. *P < 0.05, **P < 0.01, ***P < 0.001.

### Tregs lose Foxp3 expression, produce IL-17A, and become phenotypically unstable during experimental periodontitis

**Figure 4:**
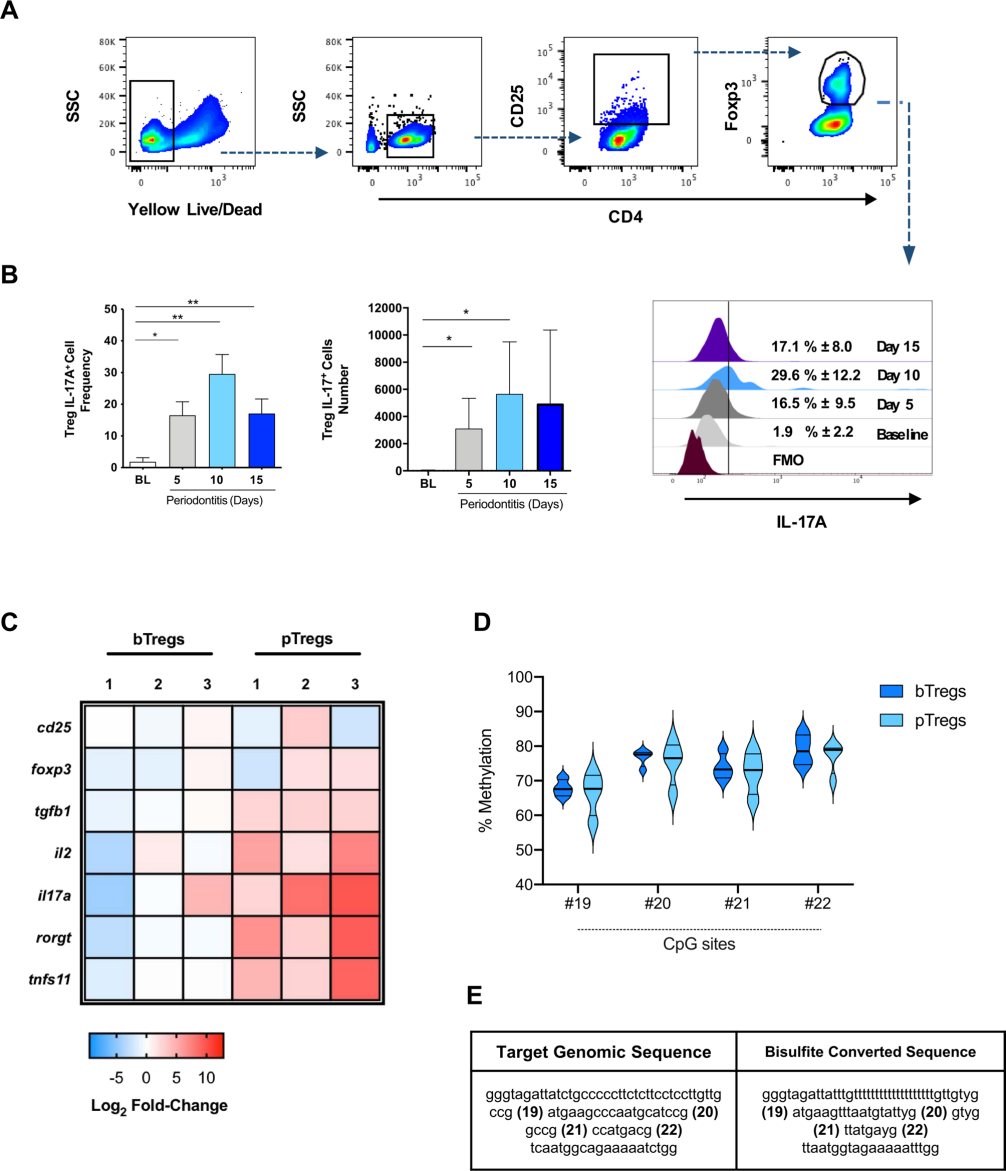
IL-17A^+^ expression, and phenotypic and epigenetic changes of Tregs from of animals with periodontitis. (**A**) Gating strategy and representative data of IL-17A expression in Tregs from cervical lymph nodes with mean percentages ± SD values. (**B**) Analysis of the frequency and total number of IL-17A^+^ Tregs during experimental periodontitis. (**C**) Heat map with the Log2 fold-change mRNA expression of Treg and Th17-related genes in isolated Tregs from periodontitis-induced animals for ten days (pTregs) or controls (bTregs), each replica includes four pooled animal samples. (**D**) Percentage of methylation of 4 CpG sites in the CNS2 zone of the *foxp3* gene in purified Tregs. (**E**) Original genomic sequence, and bisulfite conversion. FMO: Fluorescence minus one. *P < 0.05, **P < 0.01.

The flow cytometry analysis of CD4^+^CD25^+^Foxp3^+^ cells in cervical lymph nodes and spleens of animals affected with experimental periodontitis revealed significant changes in the Foxp3 expression, Treg frequency, and Treg number (Fig. 3A). We detected a sharp reduction in the Foxp3 MFI on CD4^+^CD25^+^Foxp3^+^ cells after 5- and 10-days post-periodontitis induction at all cervical lymph-nodes (≈ 28% reduction) and spleens (≈ 17% reduction) (Fig. 3B,C), although the effect was more significant in cervical lymph nodes. Also, at cervical lymph nodes, CD4^+^Foxp3^+^ cells were more frequent within the CD4^+^ compartment at day ten post-periodontitis induction (5% increase), although, at the same time point, the percentage of Foxp3^+^ cells within the CD4^+^CD25^+^ population was significantly reduced (50% less). Finally, the absolute number of CD4^+^CD25^+^Foxp3^+^ cells was higher on day ten compared to baseline and day 5 (Fig. 3A). These combined outcomes were not detected at the spleen (Fig. 3B). Considering the marked reduction in Foxp3 expression, we then assessed whether CD4^+^CD25^+^Foxp3^+^ cells could be expressing IL-17A at cervical lymph nodes (Fig. 4A). Accordingly, we detected a significant increase in the percentage of IL-17A^+^ Tregs after periodontitis induction, particularly at day 10 (p < 0.01), which correlated to their increased absolute cell number (p < 0.05) (Fig. 4B).

To assess gene expression changes in Tregs associated with periodontal disease, we isolated Tregs from healthy controls (bTregs) and periodontitis-induced animals at day 10 (pTregs). To obtain enough RNA for the qPCR assay, we pooled four animals per sample and run three replicas of each experimental group. The qPCR data revealed the increased expression of Th17-related genes (IL-17A, Rorγt, and RANKL) in Tregs isolated from diseased animals (pTregs). However, the expression of Treg-related genes was not equally affected (Fig. 4C).

Given that Foxp3 expression is regulated by epigenetic changes, as methylation of CpG sites in the *foxp3* locus, we isolated Tregs from wild-type mice affected with experimental periodontitis (10 days after periodontitis induction) and baseline controls to compare the methylation levels of 4 CpG sites at the TSDR region (Fig. 4D). Even though we did not detect significant differences between controls and diseased animals, the methylation levels of all CpG sites analyzed were higher than generally expected for Tregs (above 70% of methylation) (Fig. 4D,E). This result suggests that the expression of the Foxp3 gene in Tregs is not modulated by this epigenetic mechanism in periodontitis.

### Tregs from periodontitis-affected mice lose their suppressive capacity on osteoclastogenesis

**Figure 5:**
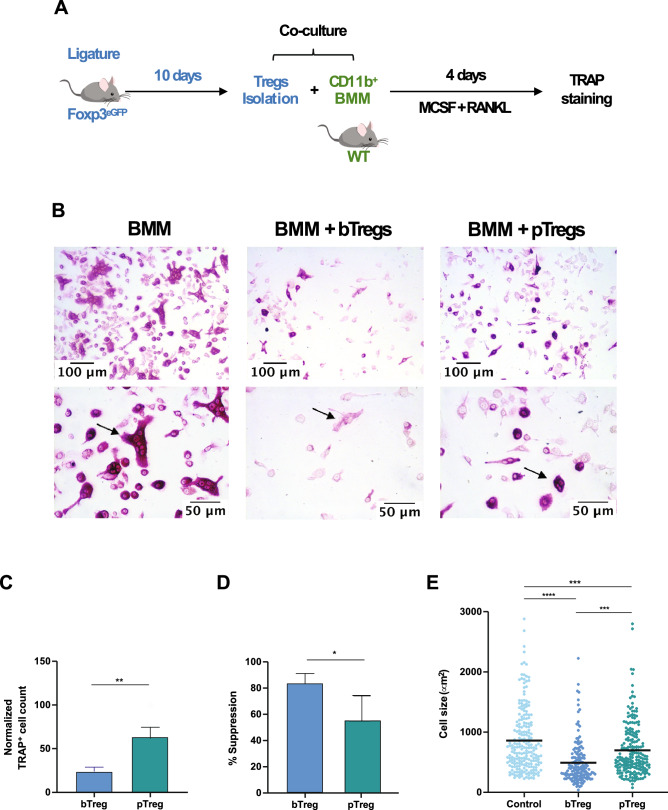
Effect of periodontitis in Treg-mediated suppression of osteoclast differentiation. (**A**) Experimental design. (**B**) Representative images of TRAP^+^ osteoclasts, arrows for a comparative view of TRAP^+^ cell size (20x and 40x). (**C**) Normalized TRAP^+^ cell count. (**D**) Percentage of suppression of osteoclast differentiation. (**E**) Cell size measured in µm^2^. BMM: Bone marrow macrophages (control), bTregs: Tregs derived from cervical lymph nodes of baseline animals, pTregs: Tregs derived from cervical lymph nodes of periodontitis-induced animals (Day 10). *P < 0.05, **P < 0.01, ***P < 0.001.

To assess the functional changes of Tregs during experimental periodontitis, in terms of their capacity to control bone-loss by directly inhibiting the differentiation of osteoclasts, we co-cultured Tregs from healthy (bTregs) and diseased (pTregs) mice (10 days after periodontitis induction) with BMM in the presence of RANKL and M-CSF (Fig. 5A). After four days, we identified and quantified the differentiation of osteoclasts using TRAP staining (Fig. 5B). We detected a significant increase in the number of osteoclasts when BMM were cultured with pTregs (*P* < 0.01) compared with those co-cultured with bTregs (Fig. 5C). This change represented a more than 30% reduction in the suppression capacity of the Tregs (Fig. 5D). We also observed a significant increase in the cell size of TRAP^+^ cells derived from the culture with pTregs compared with bTregs (*P* < 0.001), although pTreg-induced osteoclasts did not match the size of osteoclasts differentiated in the absence of Tregs (BMM) (*P* < 0.001) (Fig. 5E).

## Discussion

In the present study, we used a ligature-induced periodontitis model to assess the impact of periodontal inflammation on the phenotype and functions of cervical lymph node-derived Tregs. We also analyzed whether the Treg lineage plasticity could foster the Treg/Th17 imbalance and bone resorption characteristic of periodontitis. Tregs derived from mice with experimental periodontitis showed reduced Foxp3 expression, increased Th17-related gene expression, particularly IL-17A, and a reduced capacity to suppress osteoclastogenesis, thus indicating that periodontal inflammation affected Treg immunosuppressive capacity. Our data suggested that, although the overall number of Tregs increased during periodontitis progression, a critical number of Tregs lost their immune-suppressive properties without fully transdifferentiating into Th17-like cells. Collectively, these data demonstrated that the phenotypic stability of Tregs is critical for tissue homeostasis and regulation of inflammation.

The role of Tregs during periodontitis has been broadly documented^6,8,22^. Tregs are highly enriched in the bone-destructive form of periodontal disease -periodontitis- but not in the inflammatory but not destructive form -gingivitis- as demonstrated by the identification of CD4^+^CD25^+^CTLA-4^+^ cells and the increased expression of CTLA-4, GITR, CD103, CD45RO, and Foxp3 in gingival biopsies^23^. Accordingly, we detected a higher frequency and increased the total number of CD4^+^Foxp3^+^ cells at day ten after the induction of periodontitis. This increase was not observed in the CD4^+^CD25^+^ compartment indicating an expansion of effector T cells that highly expressed CD25 upon TCR activation^24^. When *A. actinomycetemcomitans* was used for experimental periodontitis induction, an increased number of Tregs infiltrating the gingival tissues was observed in the later stages of the infection, and Tregs prevented the infiltration of inflammatory cells and consequently suppressed bone loss^25^. These results are in line with our work, where we observed the late over-expression of Treg-related genes (Days 10 and 15) at the gingiva of animals with ligature-induced periodontitis. Although we also detected a quick decline of the Foxp3 MFI levels on Tregs at cervical lymph nodes (Day 5), suggesting that Tregs might lose the Foxp3 expression early and in parallel with the boosted Th17-type response.

Th17 lymphocytes constitute a unique effector T cell subset in periodontitis, as they are the main cellular driver of the alveolar bone loss^14^. Their development is dependent on the microbial dysbiosis and the local production of both IL-6 and IL-23^4^. Th17 cells are also the primary source of IL-17A in the gingiva, cytokine that allows the host defense against the invasion of oral bacteria^4^. IL-17A induces the expression of RANKL by different cell types during periodontitis, such as osteocytes, osteoblasts and periodontal ligament cells, which in turn increase the local net RANKL production and induced further bone loss^26–28^. Like other similar studies with ligature-induced periodontitis, we detected an early increase of transcripts of Th17-related genes at the gingival tissues and the expansion of Th17 cells in cervical lymph nodes that drain periodontal lesions^29^. Indeed, the Th17 response was associated with decreased Foxp3 expression on Tregs. Different studies have demonstrated that IL-6 has a critical role in regulating the balance between IL-17A-producing Th17 cells and Tregs^13^. IL-6, together with TGF-β, induces the development of Th17 cells from naïve T cells, whereas IL-6 in the absence of TGF-β can induce the expression of IL-17A on Tregs^30,31^. Here, we observed that among all inflammatory cytokines analyzed, IL-6 transcripts were detected at the highest level in the gingival tissues of diseased animals, five days after the placement of ligatures.

During periodontitis, IL-6 has been associated with the detection of exFoxp3 Th17 cells, a population of Th17 cells that, at one point, expressed, but ultimately lost the Foxp3 expression. These exFoxp3 Th17 cells produced high amounts of membrane-bound RANKL and were even more pathogenic than conventional Th17 cells^14^. However, the detection of IL-17A-producing cells that continue to express Foxp3 is poorly documented, although Foxp3^+^IL-17^+^ cells have been identified in periodontal lesions of patients with periodontitis^15^. Previous studies have provided cues that have suggested the phenotypical instability of Tregs. In active lesions in patients with periodontitis, Foxp3 mRNA was significantly overexpressed compared to inactive lesions, while TGF-β and IL-10 expression were downregulated in active periodontal lesions^32^. These data suggest that Foxp3-expressing cells that do not fulfill their regulatory functions through the expression of TGF-β and IL-10 could play a role in the pathogenesis of periodontitis^32^. Likewise, CD25^+^Foxp3^+^ Tregs were strikingly diminished in periodontitis lesions with bone resorption compared to healthy gingival tissues, and the correlation between RANKL and IL-10 was negative. In contrast, the correlation between RANKL and the proinflammatory cytokine IL-1β was positive^33^. Taken together, these studies implied that the production of Treg cytokines does not match the increased number of Tregs (indirectly evaluated by the Foxp3 expression), or the magnitude of the proinflammatory response.

Among the plethora of immunosuppressive functions that Tregs can display, herein, we focused on their capacity to suppress osteoclastogenesis since bone loss is the clinical hallmark of periodontitis. Tregs have an essential role in the maintenance of bone turnover under physiological and pathological conditions^34^. Indeed, when Treg functions were blocked using an anti-GITR neutralizing antibody, the levels of IL-10, CTLA-4, and TGF-β declined, accompanied by increasing bone resorption and proinflammatory cytokine production^25^. In vitro studies have shown that Tregs inhibit osteoclastogenesis from monocytes/macrophages by secreting TGF-β, IL-4, and IL-10, and by the direct interaction between CTLA-4 and CD80/86 receptors present in osteoclasts and their precursors^21,35^. In the present study, we used a similar experimental design and analyzed changes in the suppressive capacity of Tregs that were isolated from cervical lymph nodes of healthy or periodontitis animals. Tregs from periodontitis-affected mice showed a reduced suppressive capacity, which translated in an augmented detection of TRAP^+^ osteoclasts that were also larger compared to osteoclasts differentiated in the presence of Tregs obtained from healthy controls. An analogous situation has been reported in Tregs from rheumatoid arthritis patients, which secreted low levels of regulatory cytokines and had defects in the expression of CTLA-4, associated with increased bone destruction^36^.

The present study focused on the Tregs present in the cervical lymph nodes. We described the cervical lymph nodes as enlarged and enriched in Th17 cells during periodontitis. Thus, we inferred that these Tregs were exposed to the inflammatory conditions induced by the model. Here, Tregs migrate into the gingiva rather than expanding locally. Indeed, increased expression of CCL17 and CCL22 within the gingiva was associated with the infiltration of CCR4^+^ Tregs in an experimental model of periodontitis^37^. This study was followed by the gingival application of CCL22-releasing microparticles that selectively chemo-attracted Tregs to a particular periodontal lesion^37^. The technique reduced bone resorption in a canine model of periodontitis, further demonstrating that Tregs migrate into the gingival tissues from the periphery^8^. When *Porphyromonas gingivalis* (another major pathogen associated with periodontitis) was used to induce periodontitis in a K14-VEGF receptor 3-Ig (K14) mice that do not develop lymphatic vessels in the gingiva, the animals had significantly increased bone loss compared to the corresponding wild-type. Bone destruction was associated with an increased number of macrophages and MHCII^+^ antigen-presenting cells^38^. Hence, gingival lymphatics protect against *P. gingivalis*-induced periodontitis, probably through the migration of immune-suppressive cells such as Tregs. Besides, Tregs also migrate toward lymph-nodes to control antigen-presenting cells and the differentiation of effector cells. Thus, a bi-directional migration pattern is expected during inflammation^39^. In this study, we did not investigate Tregs from gingiva -infiltrating or tissue-resident- due to the technical challenge of isolating and studying them ex vivo. If such technical limitations can be resolved, gingival Treg biology may be dissected in future studies by for example a recently developed high throughput technologies that require a smaller number of cells, such as scRNAseq.

Both lymphoid and tissue-resident Tregs require the expression of Foxp3 to display their regulatory features. Foxp3 mRNA expression relies on the demethylation of the conserved non-coding sequence 2 (CNS2, also known as TSDR) in the *foxp3* locus. Natural Tregs (nTregs), produced in the thymus, possess demethylated CpG at the *foxp3* locus and show stable Foxp3 expression. TGF-β induced Tregs (iTregs), which are localized in lymph nodes, show methylated CpG, and unstable Foxp3 expression after re-stimulation, especially in the absence of TGF-β^40^. In this study, we compared the methylation of 4 CpG sites on Tregs derived from healthy or periodontitis-induced animals. In both cases, we detected high levels of methylation, consistent with iTregs. The methylation levels were more heterogeneous on Tregs derived from periodontitis, possibly due to the increased influx of Tregs and the development of newly differentiated iTregs. The overall difference between both groups was not significant, in line with the qPCR results that showed slight changes in mRNA expression of Foxp3. However, the protein levels of Foxp3 were significantly reduced, as evidenced by the sharp decrease in the Foxp3 MFI levels, followed by functionality changes. These data suggested a translational regulation of Foxp3^41^. Tregs are capable to rapidly respond to environmental cues through distinct mechanisms that regulate mRNA translation, which is advantageous since they provide a fast and energetically favorable way to shape the proteome and cell function to the extracellular context^42^. Since Foxp3 protein functions in transcriptional molecule complexes, the ensemble of such complexes is regulated by several interactions and post-translational modifications, including phosphorylation, O-GlcNAcylation, acetylation, ubiquitination, and methylation. These modifications influence each other to orchestrate Foxp3 activity and Treg suppression^42^, suggesting a novel translational regulation of Foxp3 during periodontitis.

Immunosuppressive mechanisms regulated by Tregs present a therapeutic target for complex inflammatory diseases associated with tissue destruction, as demonstrated here in an experimental model of periodontitis. Various approaches have been recently employed to increase the number of Tregs in periodontitis-affected tissues. Our data support strategies that also promote Treg functional stability rather than just increase their number. For instance, the chemoattraction of Tregs may not be enough to support their long-term functionality when Tregs infiltrate the gingival tissues, which are enriched in IL-6 and overall pro-Th17 molecular milieu. We, therefore, propose a combined concept that includes both Treg enrichment and phenotype sustainability.

## Supplementary information

Supplementary Table 1.

Supplementary Figure 1.

Supplementary Figure 2.

Supplementary Figure 3.

Supplementary Legends.
