# Supplementary material for: Regulatory T cell phenotype and anti-osteoclastogenic function in experimental periodontitis
Source: Sci Rep. 2020 Nov 4;10:19018. doi: 10.1038/s41598-020-76038-w (PMC7642388; doi:10.1038/s41598-020-76038-w)
Supplement: Supplementary file 1 — Supplementary Table 1. [file 41598_2020_76038_MOESM1_ESM.pdf]

Regulatory T cell phenotype and anti-osteoclastogenic function in experimental periodontitis

Carla Alvarez[1,2], Salwa Suliman[1,3], Rawan Almarhoumi[1], Maria Elena Vega[2], Carolina Rojas[2], Gustavo Monasterio[2], Mario Galindo[4,5], Rolando Vernal[2*], and Alpdogan Kantarci[1*]

Supplementary Table. Forward and Reverse primers used for cDNA amplifications by qPCR.

Target	***Forward Primer***	***Reverse Primer***
CD25	*TGTGCTCACAATGGAGTATAAGG*	*CTCAGGAGGAGGATGCTGAT*
CTLA-4	*GACGCAGATTTATGTCATTGCTA*	*GGCTGAAATTGCTTTTCACAT*
Foxp3	*GCACTGTGCCTGGTATATGCT*	*CTTCCAAGTCTCGTCTGAAGG*
GITR	*GAAGTCATGGCTCTTGTGCAT*	*CAGGACTCGATGGCAGTTG*
IL-10	*CAGAGCCACATGCTCCTAGA*	*TGTCCAGCTGGTCCTTTGTT*
IL-2	*GCTGTTGATGGACCTACAGGA*	*TTCAATTCTGTGGCCTGCTT*
IL-6	*GCTACCAAACTGGATATAATCAGGA*	*CCAGGTAGCTATGGTACTCCAGAA*
RANKL	*AGCCATTTGCACACCTCAC*	*AGCCATTTGCACACCTCAC*
IL-17A	*CATGAGTCCAGGGAGAGCTT*	*GCTGAGCTTTGAGGGATGAT*
IL-17F	*CCCAGGAAGACATACTTAGAAGAAA*	*CAACAGTAGCAAAGACTTGACCA*
Rorγt	*ACTGCCAGCTGTGTGCTGT*	*CATTGCCAATCCAAAGCAG*
TGF-β1	*TGGAGCAACATGTGGAACTC*	*GTCAGCAGCCGGTTACCA*
18S rRNA	*GCAATTATTCCCCATGAACG*	*GGGACTTAATCAACGCAAGC*

CTLA-4, *Cytotoxic T-Lymphocyte Antigen 4*; Foxp3, Forkhead box P3; GITR, Glucocorticoid-induced TNFR family-related gene; IL, interleukin; RANKL, receptor-activator of nuclear factor-kappa B ligand; Rorγt, nuclear receptor retinoic acid receptor-related orphan receptor gamma; TGF, tumor growth factor.
