# Supplementary material for: Regulatory T cell phenotype and anti-osteoclastogenic function in experimental periodontitis
Source: Sci Rep. 2020 Nov 4;10:19018. doi: 10.1038/s41598-020-76038-w (PMC7642388; doi:10.1038/s41598-020-76038-w)
Supplement: Supplementary file 2 — Supplementary Figure 1. [file 41598_2020_76038_MOESM2_ESM.pdf]

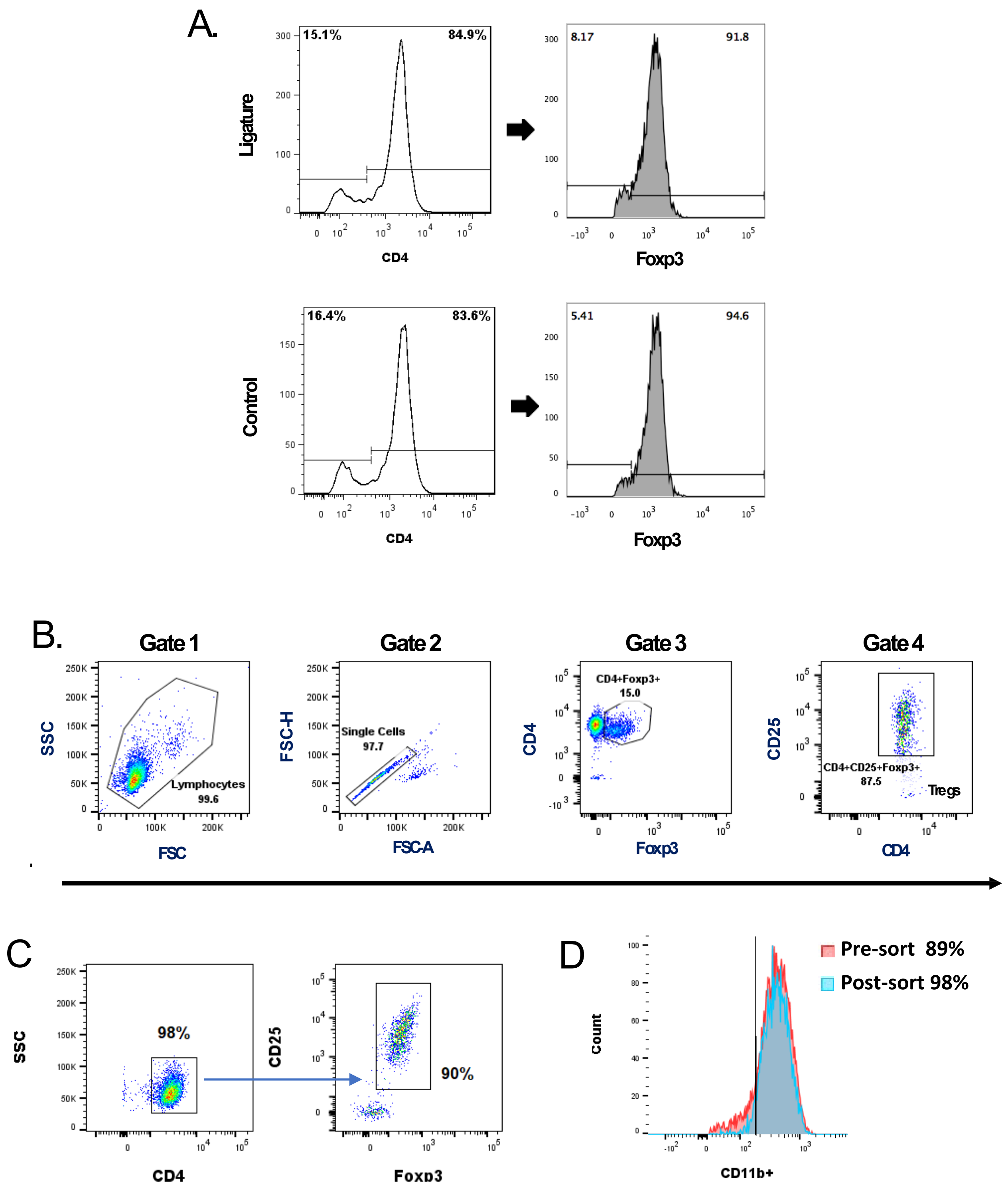

Supplementary Figure 1. Cell purification methods. A) Flow cytometry analysis of CD4$^+$CD25$^+$ cells isolated from WT animals using the microbeads-based immune-isolation system. B) Gating strategy for CD4$^+$CD25$^+$Foxp3$^+$(eGFP) cell sorting from reporter animals. C) Post-sort analysis of the CD4$^+$CD25$^+$Foxp3$^+$ cells. D) Pre and post-sort analysis of BMM derived CD11b$^+$ cells.
