# Supplementary material for: Regulatory T cell phenotype and anti-osteoclastogenic function in experimental periodontitis
Source: Sci Rep. 2020 Nov 4;10:19018. doi: 10.1038/s41598-020-76038-w (PMC7642388; doi:10.1038/s41598-020-76038-w)
Supplement: Supplementary file 3 — Supplementary Figure 2. [file 41598_2020_76038_MOESM3_ESM.pdf]

Regulatory T cell phenotype and anti-osteoclastogenic function in experimental periodontitis

Carla Alvarez[1,2], Salwa Suliman[1,3], Rawan Almarhoumi[1], Maria Elena Vega[2], Carolina Rojas[2], Gustavo Monasterio[2], Mario Galindo[4,5], Rolando Vernal[2*], and Alpdogan Kantarci[1*]

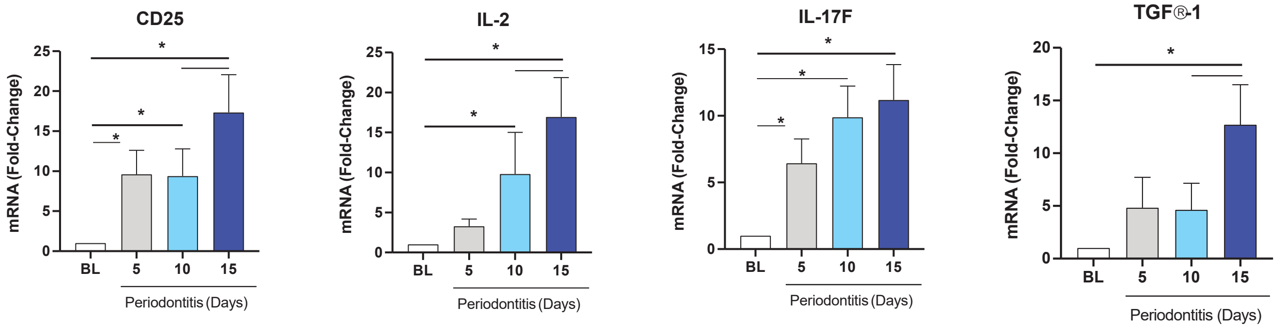

Supplementary Figure 2. Expression profile of Th17 and Tregs markers in periodontal lesions. mRNA fold-change levels of CD25, IL-2, IL-17F, and TGFβ1 in periodontal lesions of animals with ligature-induced periodontitis and control animals (BL). *P<0.05.
