# Supplementary material for: Regulatory T cell phenotype and anti-osteoclastogenic function in experimental periodontitis
Source: Sci Rep. 2020 Nov 4;10:19018. doi: 10.1038/s41598-020-76038-w (PMC7642388; doi:10.1038/s41598-020-76038-w)
Supplement: Supplementary file 4 — Supplementary Figure 3. [file 41598_2020_76038_MOESM4_ESM.pdf]

Regulatory T cell phenotype and anti-osteoclastogenic function in experimental periodontitis

Carla Alvarez[1,2], Salwa Suliman[1,3], Rawan Almarhoumi[1], Maria Elena Vega[2], Carolina Rojas[2], Gustavo Monasterio[2], Mario Galindo[4,5], Rolando Vernal[2*], and Alpdogan Kantarci[1*]

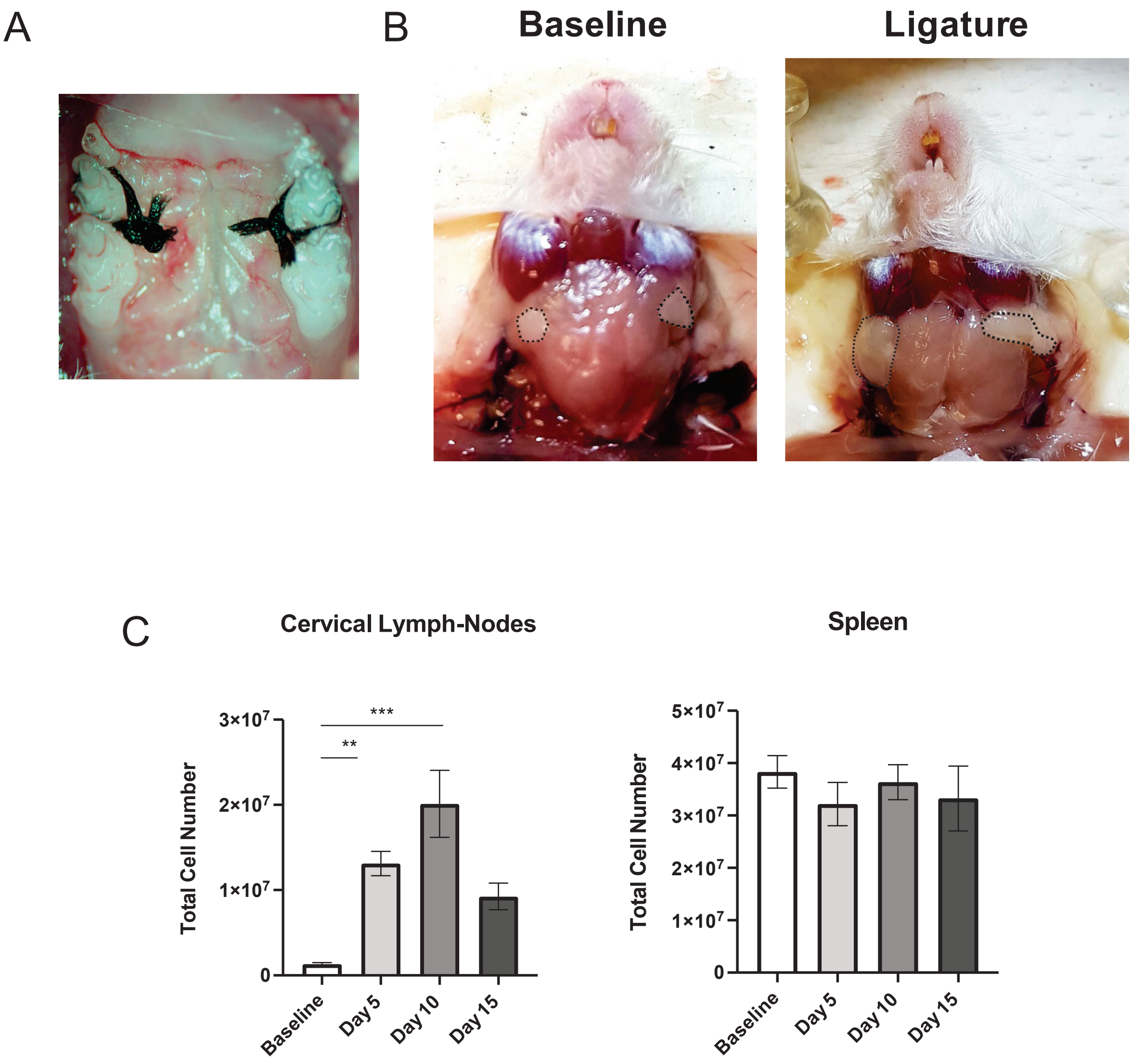

Supplementary Figure 3. Cervical Lymph-nodes size and cellularity after ligature- induced periodontitis. A) Palatal vision of the silk-ligatures tied around bilateral second molars. B) Comparison of the cervical lymph-nodes size (shown as grey area) between a baseline animal and a periodontitis-induce animal (10 days). C) Changes in the total cell number in cell suspension obtained from cervical lymph-nodes and spleens of animals with ligatures for 5, 10, or 15 days and control (Baseline).
