# Supplementary material for: Regulatory T cell phenotype and anti-osteoclastogenic function in experimental periodontitis
Source: Sci Rep. 2020 Nov 4;10:19018. doi: 10.1038/s41598-020-76038-w (PMC7642388; doi:10.1038/s41598-020-76038-w)
Supplement: Supplementary file 5 — Supplementary Legends. [file 41598_2020_76038_MOESM5_ESM.docx]

**Supplementary Table. Forward and Reverse primers used for qPCR.** CTLA-4, Cytotoxic T-Lymphocyte Antigen 4; Foxp3, Forkhead box P3; GITR, Glucocorticoid-induced TNFR family-related gene; IL, interleukin; RANKL, receptor-activator of nuclear factor-kappa B ligand; Rorγt, nuclear receptor retinoic acid receptor-related orphan receptor gamma; TGF, tumor growth factor.

**Supplementary Figure 1. Cell purification methods**. A) Flow cytometry analysis of CD4^+^CD25^+^ cells isolated from WT animals using the microbeads-based immune-isolation system. B) Gating strategy for CD4^+^CD25^+^Foxp3^+^(eGFP) cell sorting from reporter animals. C) Post-sort analysis of the CD4^+^CD25^+^Foxp3^+^ cells. D) Pre- and post-sort analysis of BMM-CD11b^+^ cells.

**Supplementary Figure 2. Expression profile of Th17 and Tregs markers in periodontal lesions.** mRNA fold-change levels of CD25, IL-2, IL-17F, and TGFβ1 in periodontal lesions of animals with ligature-induced periodontitis and control animals (BL). **P*<0.05.

**Supplementary Figure 3. Cervical Lymph-nodes size and cellularity after ligature- induced periodontitis.** A) The palatal vision of the silk-ligatures tied around bilateral second molars. B) Comparison of the cervical lymph-nodes size (shown as a grey area) between a baseline animal and a periodontitis-induce animal (10 days). C) Changes in the total cell number in cell suspension obtained from cervical lymph-nodes and spleens of animals with ligatures for 5, 10, or 15 days and control (Baseline).
